# Wearing Flannel

**Published:** 1857-11

**Authors:** 


### WEARING FLANNEL.

PUT it on at once, this first week of November, a good, substantial, old-fashioned, home-made, loose, red woollen flannel shirt, and do not lay it aside for a thinner article, at least until the first day of May, even in the latitude of New Orleans. We advise the *red*, because it does not full up, thicken, and become leathery by wearing.

Wear it only in the day-time, unless you are very much of an invalid; then change it for a similar one to sleep in—letting the two hang alternately on a chair to dry in a warm dry room. If leaving it off at night gives you a cold, never mind it; persevere until you take no more cold by the omission. No one ceases to wear shoes because they caused corns: it is the proper use of things which makes them innocuous. The less you wear at night, the more good will your clothing do you in the day-time. Those who wear a great deal of clothing at night, must wear that much more in the day, or they will feel chilly all the time; and our own observation teaches us, that the people who muffle up most are the most to complain of taking cold.

But why wear flannel next the skin, in preference to silk or cotton?

Because it is warmer; it conveys heat away from the body less rapidly; does it so slowly, that it is called a non-conductor; it feels less cold when we touch it to the skin than silk or cotton.

If the three are wetted, the flannel feels less cold at the first touch, and gets warm sooner than silk or cotton, and does not cling to the skin when damp, as much as they do. We know what a shock of coldness is imparted to the skin when, after exercise and perspiration, an Irish linen shirt worn next the skin is brought in contact, by a change of position, with a part of the skin which it did not touch a moment before—often sending a shivering chill through the whole system.

A good deal has been said and written about silk being best on account of its electrical agencies; but all that is guess-work. We are mere blind leaders of the blind when we talk about that subtle agent; and until we know more of it, it is the greater wisdom to be guided by our sensations.

Another reason why woollen flannel is better is, that while cotton and silk absorb the perspiration, and is equally saturated with it, a woollen garment conveys the moisture to its outside, where the microscope, or a very good eye, will see the water standing in innumerable drops. This is shown any hour, by covering a profusely sweating horse with a blanket, and let him stand still. In a short time, the hair and inner surface of the blanket will be dry, while the moisture will be felt on the outside. If we would be wise, we must use our senses, and observe for ourselves.

Some persons prefer white flannel, which may be prevented from fulling up, if first well washed in pretty warm soap-suds, then rinsed in one water as hot as can be well borne by the hand. After being once made, a woollen white flannel shirt should never be put in cold water, but always washed as above, not by putting soap on it, but by washing it in soap suds, not very hot.

